# Comparing LAMA with LABA and LTRA as add-on therapies in primary care asthma management

**DOI:** 10.1038/s41533-020-00205-9

**Published:** 2020-11-11

**Authors:** Alan Kaplan, J. Mark FitzGerald, Roland Buhl, Christian Vogelberg, Eckard Hamelmann

**Affiliations:** 1 Family Physician Airways Group of Canada, University of Toronto, Toronto, ON Canada; 2 Centre for Lung Health, Vancouver Coastal Health Research Institute, Vancouver, BC Canada; 3 Pulmonary Department, Johannes Gutenberg University Mainz, Mainz, Germany; 4 Department of Pediatric Pulmonology and Allergy, University Hospital Carl Gustav Carus, Technical University of Dresden, Dresden, Germany; 5 Klinik für Kinder und Jugendmedizin, Evangelisches Klinikum Bethel, Bielefeld, Germany; 6 Allergy Center of the Ruhr University, Bochum, Germany

**Keywords:** Asthma, Allergy, Asthma

## Abstract

The Global Initiative for Asthma recommends a stepwise approach to adjust asthma treatment to the needs of individual patients; inhaled corticosteroids (ICS) remain the core pharmacological treatment. However, many patients remain poorly controlled, and evidence-based algorithms to decide on the best order and rationale for add-on therapies are lacking. We explore the challenges of asthma management in primary care and review outcomes from randomised controlled trials and meta-analyses comparing the long-acting muscarinic antagonist (LAMA) tiotropium with long-acting β_2_-agonists (LABAs) or leukotriene receptor antagonists (LTRAs) as add-on to ICS in patients with asthma. In adults, LAMAs and LABAs provide a greater improvement in lung function than LTRAs as add-on to ICS. In children, results were positive and comparable between therapies, but data are scarce. This information could aid decision-making in primary care, supporting the use of add-on therapy to ICS to help improve lung function, control asthma symptoms and prevent exacerbations.

## Introduction

**Fig. 1:**
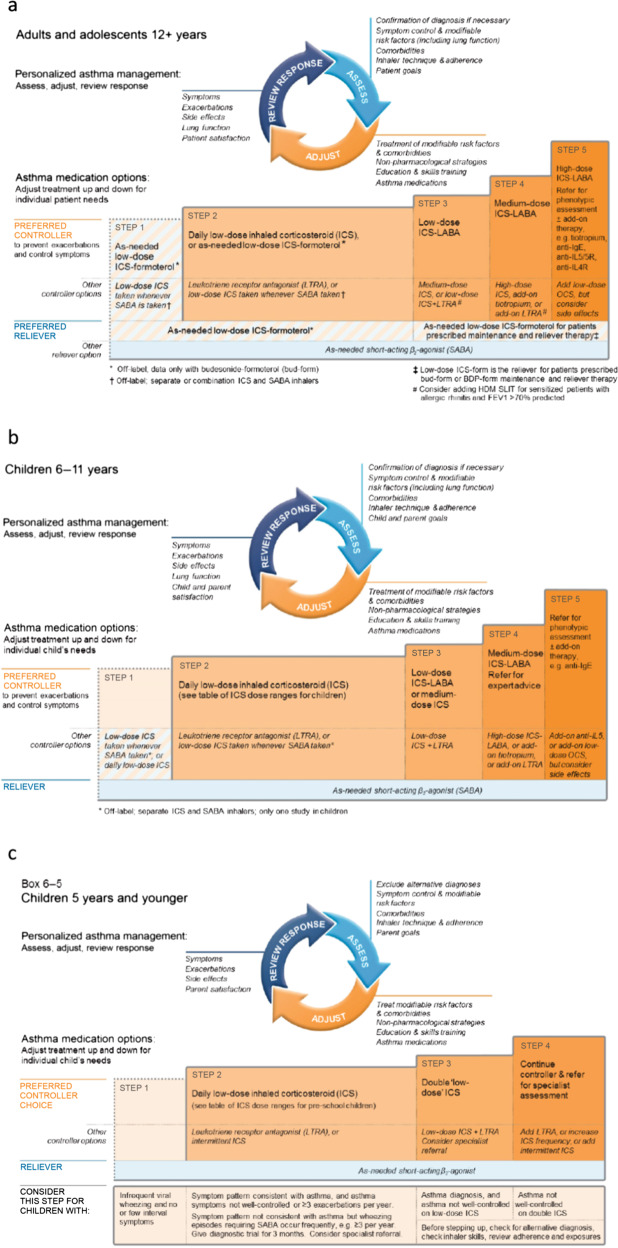
GINA treatment recommendations for patients aged ≤5 years, 6–11 years and ≥12 years^9^. © 2020, Global Initiative for Asthma, reproduced with permission. FEV_1_ forced expiratory volume in 1s, GINA Global Initiative for Asthma, ICS inhaled corticosteroid, Ig immunoglobulin, IL interleukin, LABA long-acting β_2_-agonist, LTRA leukotriene receptor antagonist, OCS oral corticosteroid, SABA short-acting β_2_-agonist.

**Fig. 2:**
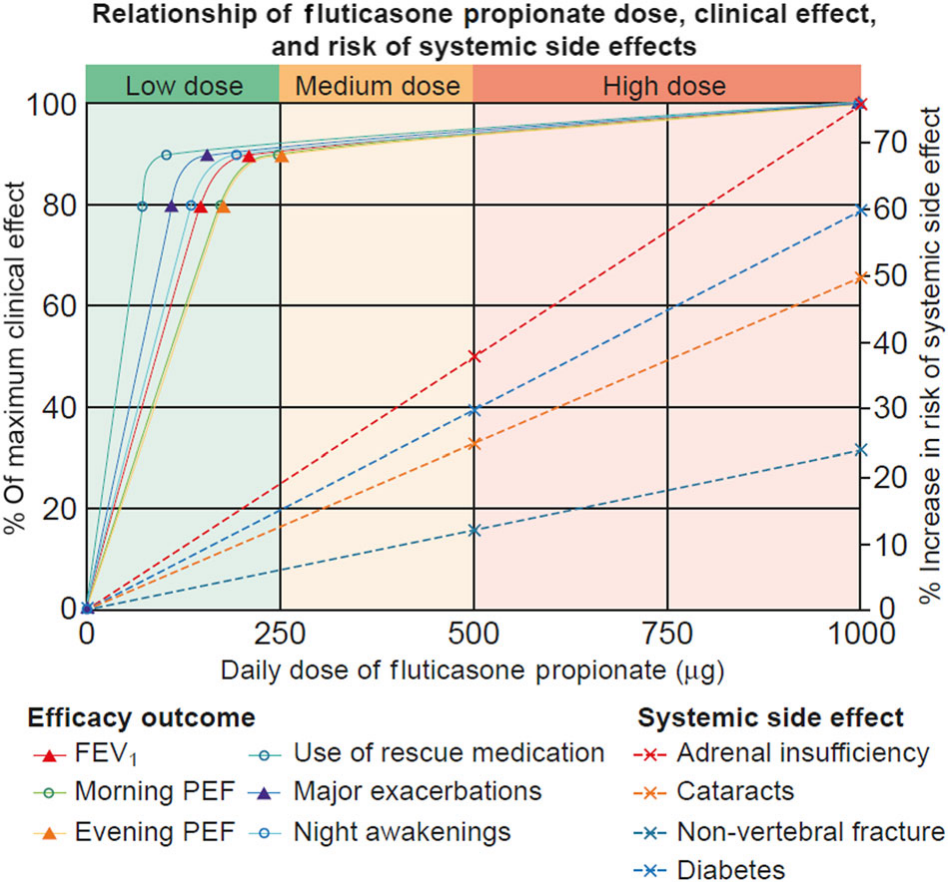
Schematic dose–response curves for different outcomes for efficacy and adverse effects with inhaled corticosteroids, expressed as fluticasone propionate in µg/day. Reprinted with permission from the American Thoracic Society. Copyright © 2020 American Thoracic Society. Beasley et al.^12^. FEV_1_ forced expiratory volume in 1 s, PEF peak expiratory flow.

Asthma is a serious global health issue that affects all age groups, with a reported 339 million sufferers worldwide, presenting a number of challenges for primary care physicians^1^. Many patients with asthma remain symptomatic, despite treatment, for multiple different reasons^2–6^. It has been suggested that patients may overestimate and thus inaccurately report their level of disease control, because they accept and tolerate a certain level of symptoms, assuming them to be an inevitable consequence of asthma^7,8^. Physicians may underestimate the prevalence and severity of symptoms and overestimate the degree to which the patient’s asthma is controlled, meaning the patient may not receive adequate medication to achieve control of their disease^2,7^. Reducing asthma symptoms and future risk through correct add-on therapy and management in patients who remain uncontrolled despite treatment is a major challenge for those working in both secondary and primary care. The Global Initiative for Asthma (GINA) strategy recommends a stepwise approach to asthma management in order to achieve symptom control and prevent future risks, including exacerbations, loss of lung function, and side effects of medication (Fig. 1)^9^. Inhaled corticosteroids (ICS) are considered an effective long-term controller treatment in the management of asthma^10^. However, if asthma remains uncontrolled despite medium-dose ICS, increasing the dose of ICS may not be appropriate due to an increased risk of local and systemic side effects and variation in individual ICS dose-responsiveness between patients. In addition, most of the clinical benefit of ICS use is seen at low doses. Add-on treatments may therefore be required^9^. In addition, evidence suggests that the ICS dose–response curve is relatively flat, with 80–90% of the maximum achievable therapeutic effect in adult asthma obtained at 200–250 μg of fluticasone propionate or equivalent (Fig. 2); therefore, addition of an add-on therapy may be considered to be a more effective and safer treatment strategy^11–13^.

Long-acting β_2_-agonist (LABA; e.g. salmeterol, formoterol, vilanterol, indicaterol^14–16^), leukotriene receptor antagonist (LTRA; e.g. montelukast and zafirlukast [discontinued]^15,17^) and long-acting muscarinic antagonist (LAMA; tiotropium^18^ [the only LAMA currently indicated for use in patients with asthma]) add-on controller therapies have been shown to improve lung function and asthma control and reduce exacerbations in asthma patients, have safety profiles similar to placebo, and are currently indicated for use in patients with asthma. More information on these classes of agents, including indications and their modes of action, is detailed in Table 1.

**Table 1:** Drug names, indications and mode of action of LABAs, LTRAs and LAMAs.

	LABA	LTRA	LAMA
Drug name(s)	Salmeterol (single and dual therapy)^a^ Formoterol (single and dual therapy)^a^ Vilanterol (dual therapy only)^a^	Montelukast Zafirlukast (discontinued)	Tiotropium (delivered via the Respimat® device)
Indication	Single therapy Patients with asthma including nocturnal asthma and exercise-induced symptoms who are treated with ICS and require a LABA in accordance with current treatment guidelines^58–61^. • Salmeterol: patients aged ≥4 years in Europe and the USA^58,59^. • Formoterol: patients aged ≥5 years in the USA and aged ≥6 years in Europe^60,61^. Dual therapy^a^ Patients with asthma where use of a LABA+ICS product is appropriate, such as in patients not adequately controlled with ICS and “as-needed” SABA or in patients already adequately controlled on both ICS and LABA. There are several dual therapies available: • Patients aged ≥4 years: Advair Diskus® (fluticasone propionate/salmeterol)^65^ • Patients aged ≥6 years: Symbicort Turbohaler® (budesonide/formoterol)^66^. • Patients aged ≥18 years: Sirdupla® (fluticasone/salmeterol), AirFluSal® (fluticasone/salmeterol), Aerivio Spiromax® (fluticasone/salmeterol), Breo Ellipta® (fluticasone furoate/vilanterol), Dulera® (mometasone/formoterol), DuoResp Spiromax® (budesonide/formoterol), Fostair® (beclomethasone/formoterol), Fobumix® (budesonide/formoterol) and Flutiform® 250/10 µg (fluticasone/formoterol)^67–75^.	• Patients with asthma aged ≥2 years with mild-to-moderate persistent asthma who are inadequately controlled on ICS and in whom SABAs provide inadequate clinical control of asthma^10,55,56^. • For patients with asthma aged ≥15 years in whom montelukast is indicated in asthma, it can also provide symptomatic relief of seasonal allergic rhinitis^10,62^. • As an alternative treatment option to low-dose ICS for patients aged 2–14 years with mild persistent asthma who do not have a recent history of serious asthma attacks that require oral corticosteroid use and who have demonstrated that they are not capable of using ICS^55,56^. • Patients aged ≥2 years in the prophylaxis of asthma in which the predominant component is exercise-induced bronchoconstriction^10,55,56^. • Montelukast oral granules are indicated in patients aged 6 months to 5 years^64^. Montelukast chewable tablets are also available for paediatric patients aged 2–14 years^51,55,56^.	In the EU: maintenance bronchodilator treatment in patients aged ≥6 years with severe asthma who experienced one or more severe asthma exacerbation in the preceding year^57^. In the USA: the long-term, once-daily, maintenance treatment of asthma in patients aged ≥6 years^63^.
Mode of action	Activate β_2_-receptors in bronchial smooth muscle, resulting in bronchodilation^34,76^	Long-term anti-inflammatory effects^14^	Induce bronchodilation through the inhibition of M_3_ receptors in bronchial smooth muscle^63^

*ICS* inhaled corticosteroid, *EU* European Union, *LABA* long-acting β2-agonist, *LAMA* long-acting muscarinic antagonist, *LTRA* leukotriene receptor antagonist, *SABA* short-acting β2-agonist.

^a^Dual therapy refers to LABA+ICS therapy administered in a single inhaler treatment.

With multiple add-on therapies available for the management of asthma, there have been several systematic reviews published that evaluate the efficacy and safety of add-on therapies compared with either placebo or another add-on therapy^14–17,19,20^. However, none compare LABA, LTRA and LAMA as add-on treatments to ICS in a single consolidated review, and there are no head-to-head trials evaluating all three treatments within the same trial. Here we systematically analyse and review the literature to explore the challenges of asthma management, the impact of poor asthma control on patients’ lives and compare outcomes from published studies. We examine the effect of three add-on treatments on lung function, asthma control, exacerbations and safety, with the aim of assisting primary care physicians in selecting the most appropriate add-on treatment to ICS.

## Results

### Search results

The literature search identified 14 relevant publications that met the inclusion criteria for this review: 2 Cochrane reviews and 12 additional randomised controlled trials (RCTs) that were not included within the Cochrane reviews.

- The search strings for LABA studies generated 164 publications, of which 1 meta-analysis and 4 additional RCTs met the criteria for inclusion in this review^16,21–23^.
- The search strings for LTRA studies generated 54 publications, of which 3 RCTs met the criteria for inclusion in this review^24–26^.
- The search strings for LAMA studies generated 106 publications, of which 8 RCTs met the criteria for inclusion in this review^21,22,27–31^.

### Asthma control in adult patients

LABA (salmeterol) significantly improved asthma control when added to ICS compared with placebo (measured by Asthma Control Questionnaire [ACQ])^21^. The LTRA (montelukast) did not have an effect on asthma control when compared with placebo (measured by ACQ) (Table 2)^32^. Data for LAMA (tiotropium) are more varied (Table 2)^21,27–31^. Paggiaro et al. reported that there was no difference of effect between tiotropium (5 µg and 2.5 µg) and placebo on ACQ score. Four papers compared the effect of LABAs directly with tiotropium. There was no significant difference between LABAs and tiotropium on asthma control as measured by ACQ (Table 2)^21,33,34^. One study included difference in Mini-Asthma Quality of Life Questionnaire (Mini-AQLQ) response scores as a secondary efficacy endpoint. At study endpoint at 16 weeks, salmeterol (50 µg) significantly improved overall Mini-AQLQ score compared with placebo, but there was no significant difference in response scores between the tiotropium (5 µg) and placebo groups. When directly compared, there was no difference in treatment response between salmeterol (50 µg) and tiotropium (5 µg) at study endpoint^22^. No studies compared asthma control, measured by ACQ, in LABA vs LTRA or tiotropium vs LTRA.

**Table 2:** ACQ-7 responder rates, exacerbations and AEs.

Reference	Drug vs comparator	Asthma control (measured as mean difference in ACQ)	Patients reporting ≥1 exacerbation	Patients reporting AEs
*LABA vs placebo*				
Paggiaro et al.^23^	Beclomethasone/formoterol fumarate 400/12 µg BID vs beclomethasone 400 µg BID	↔	✓	↔
Ducharme et al.^16^	Formoterol or salmeterol vs placebo	NR	✓	NR
Kerstjens et al.^21^	Salmeterol 50 µg BID vs placebo	✓✓	✓	↔
Bateman et al.^22^	Salmeterol 50 µg BID vs placebo	NR	✓	↔
*LTRA vs placebo*				
Djukanovic et al.^24^	Montelukast 10 mg QD vs placebo	NR	↔	NR
ALAACRC^32^	Montelukast 10 mg QD vs placebo	↔	NR	NR
Vaquerizo et al.^25^	Montelukast 10 mg QD vs placebo	NR	✓	↔
Virchow et al.^26^	Zafirlukast 80 mg BID vs placebo	NR	✓✓	↔
*LAMA vs placebo*				
Paggiaro et al.^28^	Tiotropium 5 µg QD vs placebo	↔	NR	↔
Paggiaro et al.^28^	Tiotropium 2.5 µg QD vs placebo	↔	NR	↔
Ohta et al.^29^	Tiotropium 5 µg QD vs placebo	✓	NR	↔
Ohta et al.^29^	Tiotropium 2.5 µg QD vs placebo	✓	NR	↔
Timmer et al.^31^	Tiotropium 5 µg QD vs placebo	✓✓	NR	↔
Timmer et al.^31^	Tiotropium 2.5 µg BID vs placebo	✓✓	NR	↔
Beeh et al.^30^	Tiotropium 5 µg QD vs placebo	✓✓	NR	↔
Beeh et al.^30^	Tiotropium 2.5 µg QD vs placebo	✓✓	NR	↔
Beeh et al.^30^	Tiotropium 1.25 µg QD vs placebo	✓✓	NR	↔
Kerstjens et al.^27^	Tiotropium 5 µg QD vs placebo^a^	✓	✓✓^c^	↔
Kerstjens et al.^27^	Tiotropium 5 µg QD vs placebo^b^	✓✓	✓✓^c^	↔
Kerstjens et al.^21^	Tiotropium 5 µg QD vs placebo	✓✓	✓	↔
Kerstjens et al.^21^	Tiotropium 2.5 µg QD vs placebo	✓✓	✓✓	↔
Bateman et al.^22^	Tiotropium 5 µg QD vs placebo	NR	✓	↔
*LABA vs LTRA*				
Chauhan and Ducharme^15^	Salmeterol/formoterol vs montelukast/zafirlukast	NR	✓✓	↔
*LAMA vs LABA*				
Kerstjens et al.^21^	Tiotropium 5 µg QD vs salmeterol 50 µg BID	↔	↔	↔
Kerstjens et al.^21^	Tiotropium 2.5 µg QD vs salmeterol 50 µg BID	↔	↔	↔
Wechsler et al.^34^	Tiotropium 18 µg QD vs salmeterol 50 µg or formoterol 9 µg BID	↔	↔	↔
Bateman et al.^22^	Tiotropium 5 µg QD vs salmeterol 50 µg BID	NR	↔	↔
Peters et al.^33^	Tiotropium 18 µg QD vs salmeterol 50 µg BID	↔	↔	↔

✓✓ Drug provides statistically significant improvement in outcome compared with comparator.

✓ Drug provides numerical improvement in outcome compared with comparator.

↔ Drug provides comparable outcome to comparator.

*ACQ* Asthma Control Questionnaire, *AE* adverse event, *ALAACRC* American Lung Association Asthma Clinical Research Centres, *BID* twice daily, *LABA* long-acting β_2_-agonists, *LAMA* long-acting muscarinic agonist, *LTRA* leukotriene receptor antagonist, *NR* not reported, *QD* once daily.

^a^Trial one.

^b^Trial two.

^c^Observation refers to increased time to exacerbations rather than amount.

### Lung function in adult patients

**Fig. 3:**
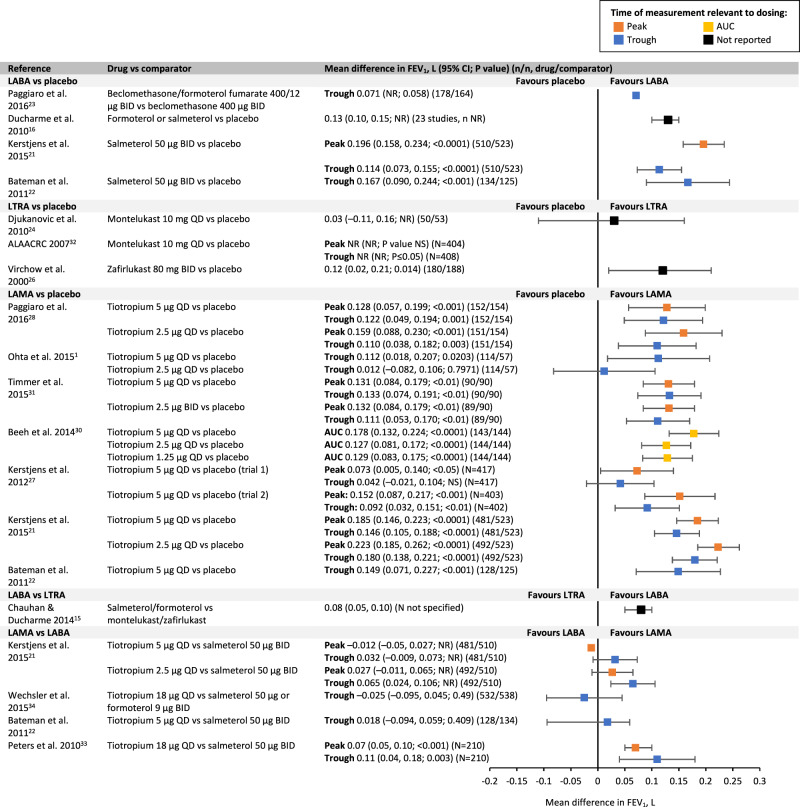
Mean difference in FEV_1_. ALAACRC American Lung Association Asthma Clinical Research Centers, AUC area under curve, BID twice daily, CI confidence interval, FEV_1_ forced expiratory volume in 1 s, LABA long-acting β_2_-agonist, LAMA long-acting muscarinic agonist, LTRA leukotriene receptor antagonist, NR not reported, NS non-significant, QD once daily.

**Fig. 4:**
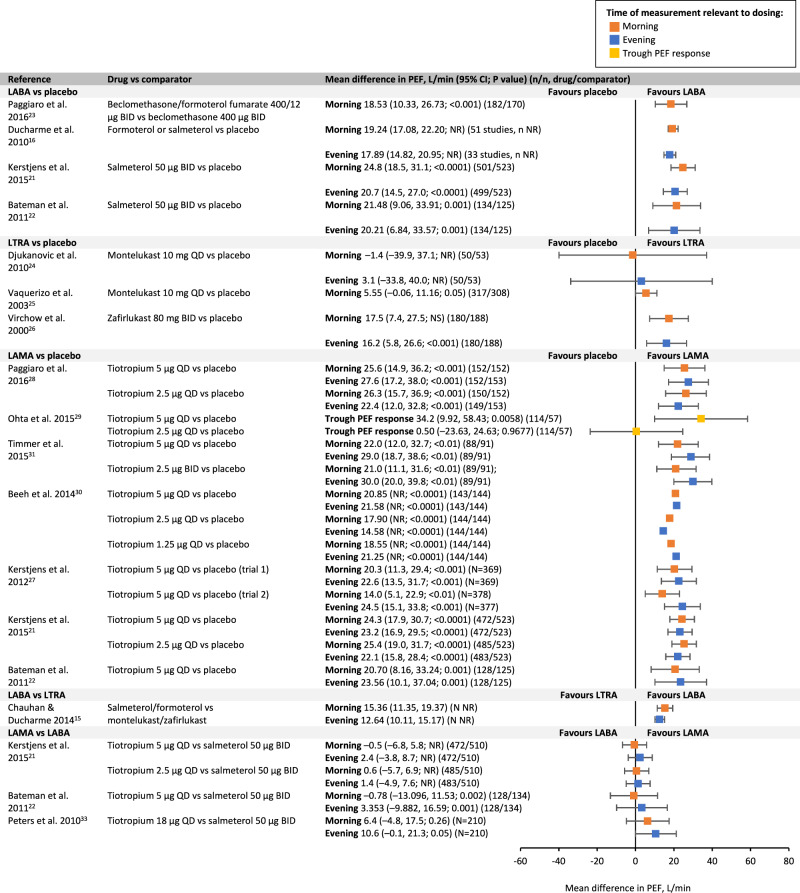
Mean difference in PEF. BID twice daily, CI confidence interval, LABA long-acting β_2_-agonist, LAMA long-acting muscarinic agonist, LTRA leukotriene receptor antagonist, NR not reported, NS non-significant, PEF peak expiratory flow, QD once daily.

**Fig. 5:**
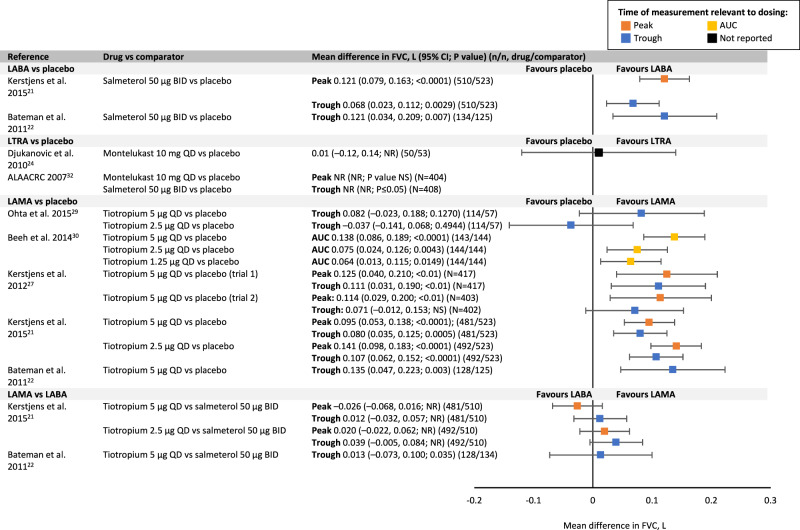
Mean difference in FVC. ALAACRC American Lung Association Asthma Clinical Research Centers, AUC area under curve, BID twice daily, CI confidence interval, FVC forced vital capacity, LABA long-acting β_2_-agonist, LAMA long-acting muscarinic agonist, LTRA leukotriene receptor antagonist, NR not reported, NS non-significant, QD once daily.

LABAs significantly improved forced expiratory volume in 1 s (FEV_1_), morning and evening peak expiratory flow (PEF) (salmeterol/formoterol) and forced vital capacity (FVC) (salmeterol) compared with placebo or ICS alone (Figs. 3–5)^16,21–23^. The literature reporting the effect of LTRAs as add-on to ICS on lung function is varied (Figs. 3–5), suggesting no beneficial effect of montelukast on FEV_1_ or FVC compared with ICS alone^24^. Results for the effect of montelukast on PEF are conflicting. Only one study of zafirlukast (now discontinued) was identified, with the authors reporting significant improvements in FEV_1_ and both morning and evening PEF compared with placebo^26^.

Of the seven available studies of LAMAs (tiotropium), all but two trial arms from two studies reported significant improvements in FEV_1_ (peak, trough and area under the curve [AUC]) and FVC compared with placebo (Figs. 3, 5)^21,22,27–31^. In all the published studies in adults^21,22,27–31^, tiotropium significantly improved morning and evening PEF compared with placebo or ICS alone, except for one tiotropium 2.5 µg trial arm, which reported no significant difference between tiotropium and placebo on trough PEF response (Fig. 4)^29^.

A systematic review comparing the use of LABAs with LTRAs as add-on to ICS reported that LABAs (salmeterol or formoterol) have a significantly greater effect on FEV_1_ and PEF compared with LTRAs (montelukast or zafirlukast) (Figs. 3, 4)^15^. There are no data available comparing the effect of LABAs and LTRAs on FVC. Four papers compared the effect of tiotropium with the LABAs salmeterol and formoterol on lung function parameters (Figs. 3, 4)^33^.

### Exacerbations in adult patients

LABAs provided numerical improvement in the number of patients reporting at least one exacerbation compared with placebo (Table 2)^21–23^. Data for LTRAs (montelukast and zafirlukast [now discontinued]) are varied (Table 2). LAMA (tiotropium) both significantly and non-significantly reduced the number of patients experiencing at least one exacerbation (Table 2)^27^. A meta-analysis comparing LABA with LTRA reported a 2% reduction in risk of exacerbations in patients using LABA+ICS vs LTRA+ICS combination therapy (Table 2)^15^. Both tiotropium and LABAs had a comparable effect on the risk of exacerbations^21,22,33,34^.

### Safety in adult patients

Overall, comparable proportions of patients report adverse events (AEs) with LABA, LTRA and tiotropium treatment as add-on to ICS with both placebo and with one another (Table 2)^15,21–23,25–31,34^.

### Efficacy and safety of LABAs, LTRAs and LAMAs in paediatric patients

A recent systematic review by Vogelberg et al. compared the efficacy and safety of LABAs, LTRAs and LAMAs (tiotropium) in paediatric patients aged 4–17 years with asthma^35^. LABA treatment as add-on to ICS improved lung function when compared with placebo, as measured by FEV_1_ and FEV_1_ % predicted. There was no difference in risk of exacerbations requiring oral corticosteroid (OCS) between LABAs plus ICS compared with ICS alone, although it should be noted that not all trials were powered to assess exacerbations. The proportion of patients experiencing AEs or serious AEs (SAEs) with the addition of LABA to ICS was broadly similar^35^. An additional RCT of 512 patients aged 5–12 years with persistent asthma reported improvements in lung function and asthma control, and no differences in risk of exacerbations and AEs, in patients receiving LABAs (formoterol) compared with those receiving placebo as add-on to ICS^36^. However, in a systematic review comparing LABA plus ICS vs higher-dose ICS in children with asthma, combination therapy led to a trend towards an increased risk of oral steroid-treated exacerbations and hospital admissions^16^.

For LTRA (montelukast), a study by Simons et al. described a greater improvement from baseline FEV_1_ in patients receiving montelukast compared with placebo^37^. In addition, a systematic review found an improvement in baseline FEV_1_ and FEV_1_ % predicted in patients receiving ICS plus montelukast compared with those receiving ICS plus placebo, but these differences were not significant^19^. There was no difference between montelukast and placebo as add-on to ICS in the risk of exacerbations^19^. Limited available data suggest that the proportion of patients experiencing AEs with the addition of montelukast to ICS is comparable with those receiving placebo as add-on to ICS^37^.

Tiotropium improved FEV_1_ and FEV_1_ % predicted as add-on to ICS with or without additional controller therapies^21,27,28,38–42^. The proportion of paediatric patients with exacerbations requiring OCS was low in all studies included within the review by Vogelberg et al.^35^. The review authors also concluded that there was no increase in the number of patients with AEs or SAEs with tiotropium compared with placebo as add-on to ICS or add-on to ICS plus other controllers^15,21–23,25–31,34^. An additional study of 102 patients aged 1–5 years with persistent asthma symptoms reported similar findings, with the number of patients reporting AEs similar in those who received tiotropium as add-on to ICS to those who received placebo as add-on^42^.

There were fewer published studies on the efficacy and safety of LABAs, LTRAs and LAMAs as add-on to ICS in patients aged <5 years compared with studies in older age groups^42–45^. An RCT of 12 patients with asthma aged 2–5 years reported that LABA (formoterol) as add-on to ICS provided rapid and sustained bronchodilation for ≥8 h compared with placebo^45^. A 12-week RCT of 689 patients with persistent asthma (≥3 episodes of asthma symptoms during the previous year) aged 2–5 years reported that LTRA (montelukast) as add-on to ICS (in at least 50% of participants) improved multiple parameters of asthma control, including daytime and overnight asthma symptoms and the percentage of days without asthma symptoms or asthma compared with placebo. There were no reported differences in the frequency of reported AEs^44^. Similarly, Bisgaard et al. reported that, in patients aged 2–5 years with intermittent asthma, montelukast significantly reduces the rate of asthma exacerbations and delayed the median time to first exacerbation compared with placebo over 12 months. However, patients in this trial did not receive montelukast as add-on to ICS^43^. A 12-week RCT of 102 children aged 1–5 years by Vrijlandt et al. reported that tiotropium as add-on to ICS with or without additional controller medications was associated with fewer reported AEs or asthma exacerbations compared with placebo. There was no significant difference in adjusted weekly mean combined daytime asthma symptom score between baseline and Week 12 between the tiotropium and placebo groups^42^.

## Discussion

The long-term aims of asthma management are symptom control, reduction of the future risk of exacerbations and airflow limitation, while at the same time minimising treatment side effects^9^. Although major advances have been made in asthma treatment and management, there still remain many patients who have poor asthma control and maintain the potential risk of worsening of their symptoms, as well as an increased risk of exacerbations, and unscheduled urgent and emergency care visits and hospitalisations^46,47^. For adults, adolescents and children, there is a need for effective add-on treatments as an alternative to increasing the ICS dose alone, as long-term, high-dose ICS use is associated with an increased risk of side effects^9^.

The findings from this literature review suggest that LABAs, LTRAs and tiotropium have similar safety profiles in both adult and paediatric populations (Table 2). Therefore, comparing the reported efficacy of the three add-on treatments in each patient population could assist with decision-making. Greater improvements in lung function have been reported with LABAs and LAMAs vs LTRAs in adults (Figs. 3–5). In addition, there appears to be greater improvements in asthma control and exacerbations with LABAs and LAMAs as add-on therapies than with LTRAs in this population.

Much of the available evidence for asthma management is based on research carried out in adults, which leads to a greater restriction of licensing of medication in children^48^, creating additional difficulties in selecting the most appropriate treatment option for paediatric patients with asthma^49^. Despite advances in care, asthma still presents a burden within this population, with many children remaining symptomatic and uncontrolled^50^. Data in patients aged <18 years are currently limited due to inherent difficulties in the study of this population; however, available evidence suggests that LABAs and tiotropium have comparable effects with respect to lung function, asthma control and exacerbations. Of the LABAs, LTRAs and LAMAs reviewed here, the LTRA montelukast is the only add-on treatment that is indicated for use in patients aged <4 years (as a chewable tablet^51^) and the only add-on treatment recommended for use in patients aged ≤5 years as an optional controller treatment^9^. Clinical trials of LTRAs in children aged ≤5 years have not demonstrated any safety concerns^52^. However, in 2020, the U.S. Food and Drug Administration (FDA) determined that a boxed warning for the LTRA montelukast was appropriate due to the risk of mental health side effects, and advised that healthcare professionals (HCPs) consider the benefits and risks of mental health side effects before prescribing montelukast^53^. Despite less published evidence regarding use of add-on therapies in paediatric patients (aged <18 years) than in adult patients (aged ≥18 years), current available data suggest that all three add-on therapies have comparable safety profiles, with LABAs and LAMAs providing greater improvements in lung function than LTRAs. When selecting the most appropriate add-on therapy for paediatric patients, it is important to consider the reported efficacy, safety data and subsequent post-marketing safety warnings (if applicable) and the indications of these add-on therapies, as not all are appropriate for all age ranges (Table 1).

When stepping up asthma therapy and considering add-on therapy, it is important to review the options available, to involve patients in decisions about their treatment and to keep a dialogue between patients and HCPs^46^. An up-to-date individualised asthma action plan can help to keep a record of any attempted treatment approaches and help the patient to self-manage^54^. The action plans should be discussed and agreed with patients and reviewed at regular intervals to make sure that they remain up to date and are fit for purpose^54^.

Poor asthma control leads to unfavourable outcomes, more frequent exacerbations, irreversible loss of lung function and even asthma-related deaths. Add-on therapy with LABA, LAMA or LTRA should be considered when asthma symptoms remain uncontrolled with at least medium–high ICS. Primary care practitioners can and should regularly assess symptom control following assessment of adherence, triggers, device technique and comorbidities. Therapy should be stepped up as recommended in GINA guidelines in order to attain optimal control, considering individual symptoms, lung function, comorbidities, inhaler technique, adherence and patient preference as important parameters for a personalised choice.

In conclusion, in adults, LAMAs and LABAs appear to provide a greater improvement in lung function than LTRAs as add-on to ICS, although there are no individual studies that directly compare LAMAs with LTRAs. LAMAs appear to be an effective alternative to LABAs for attaining asthma control, optimising lung function and preventing exacerbations, with a possible higher lung function benefit of LAMAs compared with LABAs. Data in patients aged <18 years are currently limited due to inherent difficulties in the study of this population. Current available evidence from clinical trials suggests that LABAs, LTRAs and LAMAs have comparable safety profiles, with LABAs and LAMAs providing greater improvements in lung function than LTRAs, yet it should be noted the FDA have advised a boxed warning for the LTRA montelukast due to risk of mental health side effects. Asthma should be treated in accordance with current guidelines, with regular checks made to ensure symptoms are controlled, as well as ensuring optimal strategies are in place to prevent exacerbations and achieve best lung function. If control is not achieved, treatment should be stepped up, ensuring that factors that may influence control (such as adherence, administration technique, allergic triggers and comorbidities) are addressed for each individual patient.

## Methods

Our literature search was conducted in PubMed. For the comparison of add-on therapies, we identified RCTs and meta-analyses that compared the LAMA tiotropium with LABAs or LTRAs (directly or with placebo) as add-on to ICS in patients with asthma.

Data from RCTs of ≥4 weeks’ duration in all patients with asthma of all age groups, reporting change in FEV_1_, asthma control, exacerbations and AEs were included. Data were extracted from published manuscripts and publicly available online data. We checked the reference lists of the systematic reviews for references with any additional data for endpoints that were not described in the systematic reviews and to ensure that all trials met the inclusion criteria^77–116^. Search strings are detailed in Supplementary Methods.

### Reporting summary

Further information on research design is available in the Nature Research Reporting Summary linked to this article.

## Supplementary information

Supplementary Information

Reporting Summary
