# Supplementary material for: Comparing LAMA with LABA and LTRA as add-on therapies in primary care asthma management
Source: NPJ Prim Care Respir Med. 2020 Nov 11;30:50. doi: 10.1038/s41533-020-00205-9 (PMC7658210; doi:10.1038/s41533-020-00205-9)
Supplement: Supplementary file 1 — Supplementary Information [file 41533_2020_205_MOESM1_ESM.pdf]

Comparing LAMA with LABA and LTRA as add-on therapies in primary care asthma management

Alan Kaplan,[1*] J. Mark FitzGerald,[2] Roland Buhl,[3] Christian Vogelberg,[4] Eckard Hamelmann[5]

[1]Family Physician Airways Group of Canada, University of Toronto, Toronto, ON, Canada; [2]Centre for Heart and Lung Health, Vancouver, BC, Canada; [3]Pulmonary Department, Johannes Gutenberg University Mainz, Mainz, Germany; [4]Department of Pediatric Pulmonology and Allergy, University Hospital Carl Gustav Carus, Technical University of Dresden, Dresden, Germany; [5]Klinik für Kinder und Jugendmedizin, Evangelisches Klinikum Bethel, Bielefeld, and Allergy Center of the Ruhr University, Bochum, Germany

SUPPLEMENTARY METHODS

Search strings

Studies of LABA as add-on to ICS

((((((((clinical trial[MeSH Terms]) OR clinical trial) OR clinical study)))))

AND asthma[MeSH Terms]))

AND (((((((Asthma Control Questionnaire) OR ACQ)) OR ((forced expiratory volume) OR FEV)) OR ((exacerbation) OR worsening)) OR adverse event)))))

AND ((((((((seretide) OR symbicort) OR advair) OR viani) OR flutiform))

OR ((((((((((glucocorticoids[MeSH Terms]) OR inhaled corticosteroid*) OR budesonide) OR beclomethasone) OR beclometasone) OR fluticasone) OR triamcinolone) OR flunisolide) OR ciclesonide)) AND ((((((((adrenergic beta 2 receptor antagonists[MeSH Terms]) OR ((((beta*) AND agonist*)) AND ((long-acting) OR “long acting”))) OR ((((beta*) AND adrenergic*)) AND ((long-acting) OR “long acting”))) OR ((bronchodilat*) AND ((long-acting) OR “long acting”))) OR salmeterol) OR serevent) OR *formoterol) OR foradil) OR vilanterol))))))

AND ("2015/05/01"[Date - Publication]: "2020/02/19"[Date - Publication])

Studies of LTRA as add-on to ICS

((((((((clinical trial[MeSH Terms]) OR clinical trial) OR clinical study)))))

AND asthma[MeSH Terms]))

AND (((((((Asthma Control Questionnaire) OR ACQ)) OR ((forced expiratory volume) OR FEV)) OR ((exacerbation) OR worsening)) OR adverse event)))))

AND ((((((((((((glucocorticoids[MeSH Terms]) OR inhaled corticosteroid*) OR budesonide) OR beclomethasone) OR beclometasone) OR fluticasone) OR triamcinolone) OR flunisolide) OR ciclesonide))) AND (((((((((((leukotriene antagonists[MeSH Terms]) OR LTRA) OR leukotriene*) OR leucotriene*) OR anti-leukotriene*) OR anti-leucotriene*) OR montelukast) OR singulair) OR zafirlukast) OR accolate) OR pranlukast) OR azlaire))))

AND ("2013/01/01"[Date - Publication] : "2020/02/19"[Date - Publication])

Studies of LAMA as add-on to ICS

((((((((clinical trial[MeSH Terms]) OR clinical trial) OR clinical study)))))

AND asthma[MeSH Terms]))

AND (((((((Asthma Control Questionnaire) OR ACQ)) OR ((forced expiratory volume) OR FEV)) OR ((exacerbation) OR worsening)) OR adverse event)))))

AND ((((((((((((glucocorticoids[MeSH Terms]) OR inhaled corticosteroid*) OR budesonide) OR beclomethasone) OR beclometasone) OR fluticasone) OR triamcinolone) OR flunisolide) OR ciclesonide))) AND (((((((((((((((((((((muscarinic) AND antagonist*)))) AND (((long-acting) OR “long acting”)))))) OR ((antagonists, muscarinic[MeSH Terms]) AND (((long-acting) OR “long acting”)))))) OR LAMA) OR glycopyrronium) OR aclidinium) OR tiotropium) OR umeclidinium) OR NVA237) OR seebri) OR LAS34273) OR turdorza) OR pressair) OR eklira) OR genuair) OR spiriva) OR GSK573719))))

AND ("2015/05/01"[Date - Publication] : "2020/02/19"[Date - Publication])
